# BREATHING LIFE INTO CONVERSATIONS ON DEATH: USING A DEATH CAFÉ MODEL IN A SERVICE-LEARNING PROJECT

**DOI:** 10.1093/geroni/igac059.1715

**Published:** 2022-12-20

**Authors:** Marjorie Getz

**Affiliations:** Methodist College, Peoria, Illinois, United States

## Abstract

This poster presentation describes a project from an upper-level course on death and dying (part of a gerontology program at a small Midwest college). The course derives from the work of Phillipe Ariès’s book, The Hour of Our Death. Ariès traced five patterns across time and showed why death had become an uncomfortable truth for those of us living now. In essence, death has been banished from our daily lives. Other research suggests that younger healthcare professionals are particularly uncomfortable interacting with dying clients and may withdraw from them at this important life juncture. Finally, frustration with textbook development of cultural approaches to dying and death (often varied cultural groups, e.g., Asian Americans, are lumped together as if all Asian-Americans share death related practices) led to design of this unique service-learning project. Students, and community members who are experts on various faith/cultural practices take part in a modified death café. Students from an OLLI program are invited to participate, too. We report on qualitative data taken from student reflections. Content analyses of reflective essays identified these themes: (a) new insights/questions about death encounters/attitudes/practices in America (b) willingness to assimilate and accommodate various aspects of other cultural approaches to death into one’s own philosophy; (c) feelings of accomplishment/awareness of new skills in providing community-based support to people isolated during the pandemic; (d) understandings related to the importance/value of community service and civic engagement; and (e) new found comfort in openly discussing death concepts in this supportive environment.

